# NF-κB feedback control of JNK1 activation modulates TRPV1-induced increases in IL-6 and IL-8 release by human corneal epithelial cells

**Published:** 2011-12-02

**Authors:** Z. Wang, Y. Yang, H. Yang, J.E. Capó-Aponte, S.D. Tachado, J.M. Wolosin, P.S. Reinach

**Affiliations:** 1 Department of Biological Sciences, State University of New York, State College of Optometry, New York, NY; 2 Visual Sciences Branch, U.S. Army Aeromedical Research Laboratory, Fort Rucker, AL; 3 Department of Ophthalmology and the Black Family Stem Cell Institute, Mount Sinai School of Medicine, New York, NY; 4 Department of Medicine, Beth Israel Deaconess Medical Center and Harvard Medical School, Boston, MA

## Abstract

**Purpose:**

The corneal wound healing response to an alkali burn results in dysregulated inflammation and opacity. Transient receptor potential vanilloid type1 (TRPV1) ion channel activation by such a stress contributes to this unfavorable outcome. Accordingly, we sought to identify potential drug targets for mitigating this response, in human corneal epithelial cells (HCEC).

**Methods:**

SV40-immmortalized HCEC were transduced with lentiviral vectors to establish stable c-Jun N-terminal kinase1 (JNK1), nuclear factor-κB1 (NF-κB1), and dual specificity phsophatase1 (DUSP1) shRNAmir sublines. Immunoblotting evaluated the expression of NF-κB1, DUSP1, protein kinase Cδ (PKCδ), and the phosphorylation status of cell signaling mediators. Enzyme-linked immunosorbent assay (ELISA) evaluated interleukin-6 (IL-6) and interleukin-8 (IL-8) release.

**Results:**

Capsaicin (CAP; a selective TRPV1 agonist), induced time-dependent activation of transforming growth factor-activated kinase 1 (TAK1) and mitogen-activated protein kinase (MAPK) cascades temporally followed by increased nuclear factor of kappa light polypeptide gene enhancer in B-cells inhibitor, alpha (IκBα) phosphorylation, rises in both PKCδ protein levels and IL-6 and IL-8 release. All of these responses were blocked by the TAK1 inhibitor 5z-7-oxozeaenol (5z-OX). In the JNK1 subline, CAP failed to increase IL-6/8 release, but still stimulated NF-κB by 50%. In the NF-κB1 subline, these IL-6/8 responses were absent, JNK1 activation was attenuated and there was a concomitant increase in DUSP1 expression compared to the control. In the DUSP1 subline, JNK1 phosphorylation was enhanced and prolonged and accompanied by larger increases in IL-6/8 release.

**Conclusions:**

TRPV1 induced increases in IL-6/IL-8 release occur through TAK1 activation of JNK1-dependent and JNK1-independent signaling pathways. Their joint activation is required for NF-κB to elicit sufficient positive feedback control of JNK1/2 phosphorylation to elicit increases in IL-6/8 release. Such regulation depends on NF-κB modulation of DUSP1 expression levels and associated changes in PKCδ protein levels.

## Introduction

Severe corneal injury by an alkali burn leads to dysregulated inflammatory responses and scarring during wound healing. Recent studies show that transient receptor vanilloid type1 (TRPV1) channel activation by endogenous vanilloids and endocannabinoids may play a critical role in this sight-compromising outcome [1]. TRPV1, originally identified as the receptor for the pungent chili pepper component capsaicin (CAP), acts as an integrator for noxious thermal and chemical stimuli to transduce pain and inflammation in a host of different tissues [2,3]. Accordingly, extensive effort is being exerted to develop TRPV1-related therapeutic strategies to mitigate these stress-induced responses.

Outcomes of TRPV1 activation in human corneal epithelial cells (HCEC) include enhanced release of interleukin-6 (IL-6), a proinflammatory agent and interleukin-8 (IL-8), a chemoattractant [4-6]. Identified signal transduction events mediating these responses include transient intracellular Ca^2+^ rises and phosphorylation of kinases belonging to the p38, extracellular regulated kinase (ERK)1/2 and c-jun terminal kinase (JNK)1/2 mitogen-activated protein kinase (MAPK) cascades suggesting that all three MAPK pathways may be involved in downstream TRPV1 effects. In the same cells, however, inflammatory responses mediated by Toll-like receptor (TLR) have been recently reported to depend solely on the JNK MAPK pathway leading to nuclear factor-κB (NF-κB) activation. The physiologic relevance of JNK activation was documented by showing in JNK1^−/−^ (knockout) mice that TLR2-induced corneal stromal neutrophil recruitment and haze development were markedly reduced [7]. These aforementioned results prompted us to examine the roles of the different MAPK pathways and other relevant proteins on IL-6/8 release when mediated instead by TRPV1.

We show here in HCEC that: a) CAP induces cytokine release through sequential activation of transforming growth factor-activated kinase 1 (TAK1), JNK1 and NF-κB; b) NF-κB activation is mediated by TAK1 through both JNK1-dependent and JNK1-independent pathways; c) NF-κB contributes to JNK1 activation through a positive feedback control; d) This feedback is mediated through modulation of dual specific protein phosphatase 1 (DUSP1) expression levels. These findings suggest that DUSP1 and JNK1 are novel potential drug targets for suppressing injury-induced inflammation and scarring associated with TRPV1 activation.

## Methods

### Reagents

Capsaicin (CAP), capsazepine (CPZ), TAK1 inhibitor 5z-7-oxozeaenol (5z-OX), and pyrrolidinedithiocarbamate (PDTC) were purchased from Sigma-Aldrich (St. Louis, MO). Anti-phospho-ERK, total-ERK, total-p38, total-JNK, and β-actin antibodies were from Santa Cruz Biotechnology (Santa Cruz, CA). Anti-phospho-TAK1, phospho-p38, phospho-JNK/SAPK, phospho-nuclear factor of kappa light polypeptide gene enhancer in B-cells inhibitor, alpha (IκBα), total-TAK1, total-NF-κB1 and protein kinase Cδ (PKCδ) antibodies were purchased from Cell Signaling Technology (Danvers, MA). The anti- DUSP1 antibody was obtained from ABNOVA (Walnut Creek, CA). IL-6 and IL-8 ELISA kits were from R&D Systems (Minneapolis, MN).

### Cell culture

SV40 adenovirus-immortalized HCEC, a generous gift from Araki-Sasaki (Kagoshima Miyata Eye Clinic, Kagoshima, Japan), were cultured at 37 °C in the presence of 5% CO_2_, 95% atmosphere air in a humidiﬁed incubator with 1:1 mix of Dulbecco’s modiﬁed Eagle’s medium and Ham F12 (DMEM/F12) supplemented with 10% fetal bovine serum (FBS), 5 ng/ml EGF, 5 μg/ml insulin, and 40 μg/ml gentamicin. CAP-induced responses were elicited subsequent to overnight serum starvation in growth factor-free medium.

### Lentiviral vectors

Lentiviral vectors for stable expression of shRNAmir sequences against DUSP1, JNK1, and NF-κB1 were generated using pGIPz plasmid clones V3LHS_352110, V2LHS_61931, and V3LHS_170502, respectively (Open Biosystems, Hunstville, AL). Blast analysis of the 20-mer antisense sequences expressed by these clones demonstrated that in each case only the intended target RNA was a full sequence match. The expression cassette incorporated into the host cell DNA by these lentiviral vectors drives the expression of a turbo-GFP protein, the shRNAmir sequence and a puromycin selection gene from a single CMV promoter. Viral particles were generated by transducing HEK293T cells cultured in 100-mm dishes with 2 μg active plasmid and 8 μg of packing plasmid mix (Biogenova UMIX™; Potomac, MD) in 10 ml medium (DMEM with 10% FBS) containing 20 μl HEKFectin (BioRad, Richmond, CA). The culture medium was refreshed after overnight incubation and virus-rich supernatant was collected 48 h later after confirming that most HEK293 cells developed strong GFP fluorescence. Transduced cells were selected with puromycin (5 μg/ml) using the disappearance of all GFP-negative cells and elimination of all cells from an untransduced control culture as selection end points. To generate a control subline for evaluating any non-specific effects of shRNAmirs, viral particles incorporated a cassette for the simultaneous expression of a non-coding shRNAmir and puromycin resistance. After puromycin selection, these cells were used to determine if transfection had non-specific effects on cell-line functional properties.

### Western blot analysis

After HCEC reached about 80% confluence, they were gently washed twice in cold phosphate-buffered saline (PBS) and harvested in 0.5 ml cell lysis buffer containing 20 mM Tris, 150 mM NaCl, 1 mM EDTA, 1 mM EGTA, 1% Triton X-100, 2.5 mM sodium pyrophosphate, 1 mM β-glycerol phosphate, and 1 mM Na_3_VO_4_, pH 7.5, with a protease inhibitor mixture (1 mM PMSF, 1 mM benzamidine, 10 μg/ml leupeptin, and 10 μg/ml aprotinin). Cells were scraped with a rubber policeman, followed by sonication and centrifugation (10,000× g for 15 min at 4 °C). The protein concentration of each lysate was determined by bicinchoninic acid assay (micro BCA protein assay kit; Pierce Biotechnology, Rockford, IL). After boiling samples for 5 min, 20–50 μg denatured protein was electrophoresed in 10% polyacrylamide sodium dodecylsulphate (SDS) minigels, followed by electrophoresis and blotted onto an Immun-Blot PVDF membranes (Bio-Rad, Hercules, CA). Membranes were blocked with blocking buffer, 5% fat-free milk in 1× PBS buffer-0.1% Tween-20 for 1 h at room temperature reacted overnight at 4 °C and probed with primary antibodies of interest. Membranes were incubated with 1:2,000 dilution of a secondary antibody with goat anti-rabbit or mouse IgG for 1 h at room temperature. Immunobound antibody was visualized using an enhanced chemiluminescence detection system ECL Plus (GE Healthcare, Piscataway, NJ). Images were analyzed by densitometry (SigmaScan Pro Mountin View, CA). All experiments were repeated at least three times unless otherwise indicated.

### Enzyme-linked immunosorbent assay (ELISA)

ELISA for IL-6 and IL-8 was performed according to the manufacturer’s instructions. The amount of IL-6 or IL-8 in the culture medium was normalized to total amount of protein obtained by dissolving the washed cells in 2% SDS and 0.5 N NaOH. Each experiment was performed in triplicate with three replicates.

### Data analysis

All results are reported as means±SEM. Statistical significance was assessed using the using non-paired Students *t*-test. They were considered significant if p<0.05.

## Results

### TRPV1-induces IL-6/8 release through TAK1 and MAPK phosphorylation

**Figure 1:**
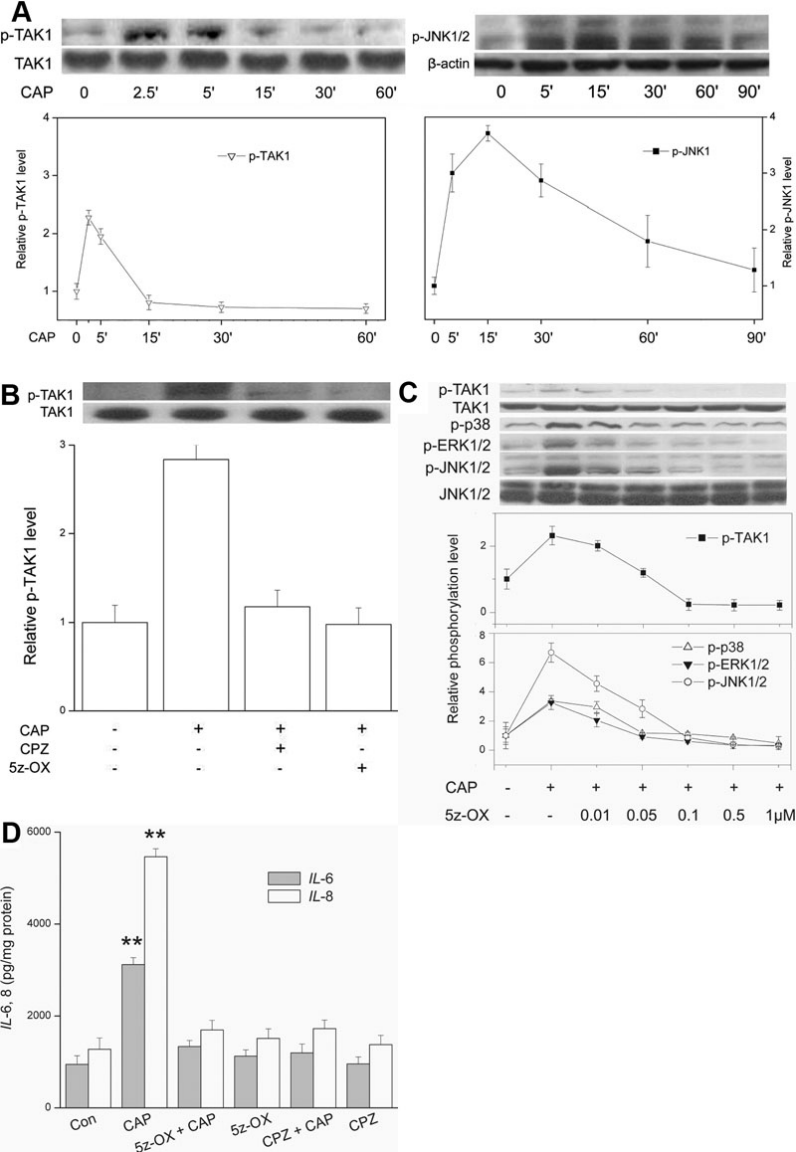
CAP induces TAK1 activation. **A**: TRPV1-induced TAK1 and JNK1 phosphorylation. CAP (20 µM) in scrambled shRNA HCEC subline caused time-dependent changes in TAK1 (left) and JNK1 (right) phosphorylation as revealed by western blot analysis. TAK1 expression level invariance validates protein loading equivalence. Results shown are representative of three independent experiments. **B**: Inhibition of TAK1 phosphorylation. Preincubation of scrambled shRNA subline for 60 min with either CPZ (10 µM) or 5z-OX (0.1 µM) suppressed CAP (20 µM)-induced TAK1 activation. TAK1 expression level invariance validates protein loading equivalence. **C**: Dose-dependent inhibitory effects of 5z-OX on TAK1 and MAPK phosphorylation. Scrambled shRNA subline was preincubated with 5z-OX (0.01–1.0 µM) for 1 h before exposure to CAP (20 µM) for 5 min. JNK1/2 expression level invariance validates protein loading equivalence. **D**: Dependence of IL-6 and IL-8 increases on TRPV1 and TAK1 activation. ELISA was performed after 24 h in presence or absence of CAP (20 µM). Each experiment was performed three times using triplicate samples. Results are expressed in pg/mg protein. Following 60 min exposure to CPZ (10 µM) or 5z-OX (0.1 µM), the effects of CAP (20 µM) were determined on the release of IL-6 and IL-8.

In Figure 1A (left and right panels), the time-dependent effects in the scrambled shRNA subline are compared of 20 μM CAP addition on phosphorylated-TAK1 (p-TAK1) and p-JNK1 formation. With TAK1, its activation level increased by 1.3 fold after 2.5 min followed by a decline after 30 min to reach nearly a baseline value. On the other hand, increases in JNK1 phosphorylation status occurred with a slower course reaching after 15 min a level that was nearly threefold above the control. Figure 1B shows that either 10 μM CPZ (a TRPV1 antagonist) or 5z-OX (a selective TAK1 inhibitor) suppressed the peak rise by 90% and 100% respectively indicating that TAK1 activation is indeed mediated by TRPV1. Figure 1C,D show that in addition to its effect on TAK1, 1 μM 5z-OX fully inhibited CAP induced phosphorylations of all three MAPK terminal kinases and blocked the CAP-induced rises in IL-6 and IL-8 release without suppressing baseline levels of IL-6/8 release. CPZ also did not affect changes in their baseline values showing that this antagonist was not cytotoxic.

### TRPV1-induced IL-6/8 release requires activation of both JNK1 and NF-κB

**Figure 2:**
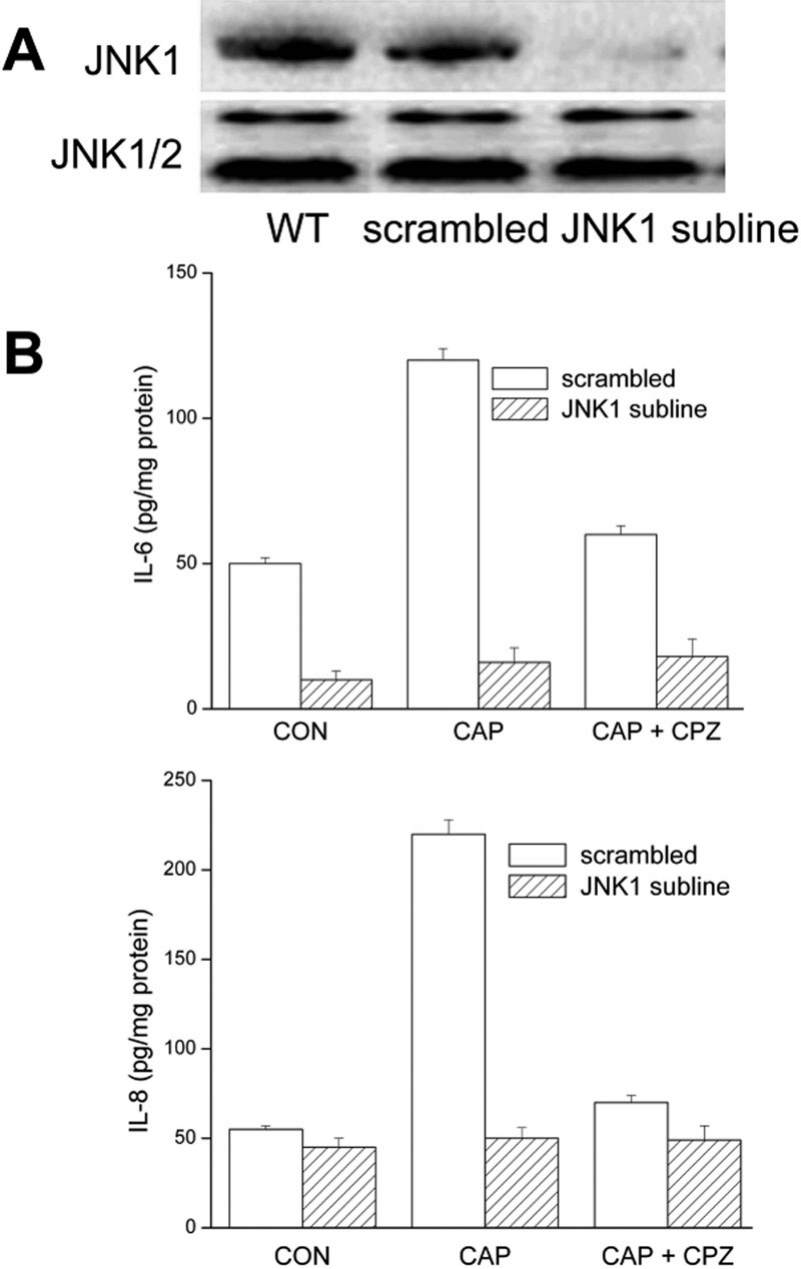
Dependence of CAP-induced increases in IL-6 and IL-8 release on JNK1 activation. **A**: Validation of JNK1 knockdown. Western blot analysis compares total JNK1 protein expression in parental wildtype (WT), resting scrambled shRNA and JNK1 HCEC subline. Expression levels in scrambled and WT HCEC were the same. JNK1/2 expression level invariance validates protein loading equivalence. **B**: Loss of JNK1 expression obviates IL-6/8 responses to TRPV1 activation. ELISA was performed on scrambled shRNA and JNK1 subline after 24 h in the presence or absence of CAP (20 µM). Results are from three independent experiments each performed in triplicate.

The role of JNK1 activation in mediating CAP-induced increases in IL-6/8 was determined in the JNK1 subline. JNK1 protein levels in this subline were reduced by more than 90% (Figure 2A) relative to the scrambled shRNA cell line and were indistinguishable from those in the parent HCEC. The drastic decrease in JNK1 expression abolished CAP-induced increases in IL-6/8 release (Figure 2B) demonstrating the central role of JNK1 activity in driving these responses. The selectivity of the shRNA design in suppressing JNK1 expression was previously validated by showing in the JNK1 shRNA subline that the mitogenic response to EGF was enhanced relative to that in scrambled cells [8].

**Figure 3:**
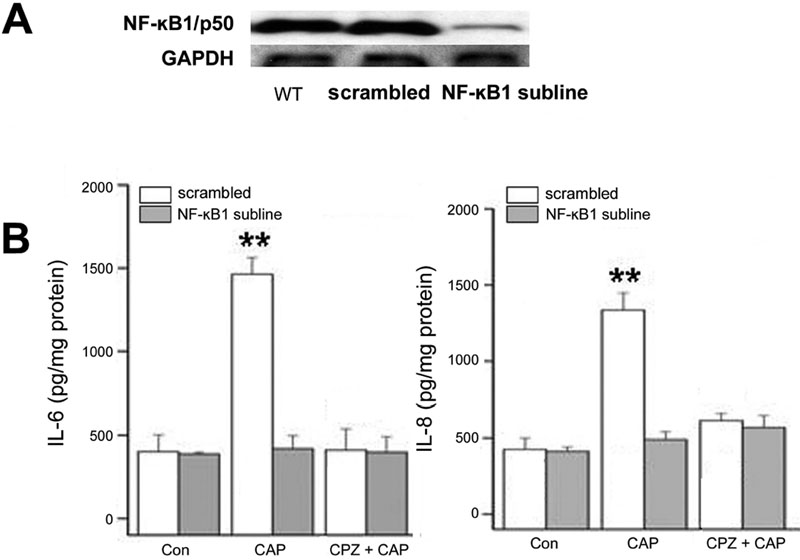
Dependence of TRPV1-induced increases in IL-6/8 release on NF-κB activation. **A**: NF-κB1/p50 protein expression levels in parental WT cells, scrambled shRNA and NF-κB sublines were compared by western blot analysis. Glyceraldehyde-3-phosphate dehydrogenase (*GAPDH*) expression level invariance validates protein loading equivalence. **B**: CAP fails to induce IL-6 and IL-8 rises in NF-κB1 subline. ELISA was performed after 24 h in the presence or absence of CAP (20 µM) for both scrambled shRNA and JNK1 sublines. Results are from three independent experiments each performed in triplicate.

A NF-κB1 subline assessed NF-κB involvement in eliciting CAP-induced IL-6/8 release. Figure 3A shows that the knockdown was nearly complete. As CAP failed to cause any increases in IL-6 /8 release in this subline, this negative effect demonstrates the essential role of this inflammation mediator in the TRPV1 response (c.f. Figure 3B [left and right]).

### TAK1 and JNK1 activities are essential for NF-κB activation

**Figure 4:**
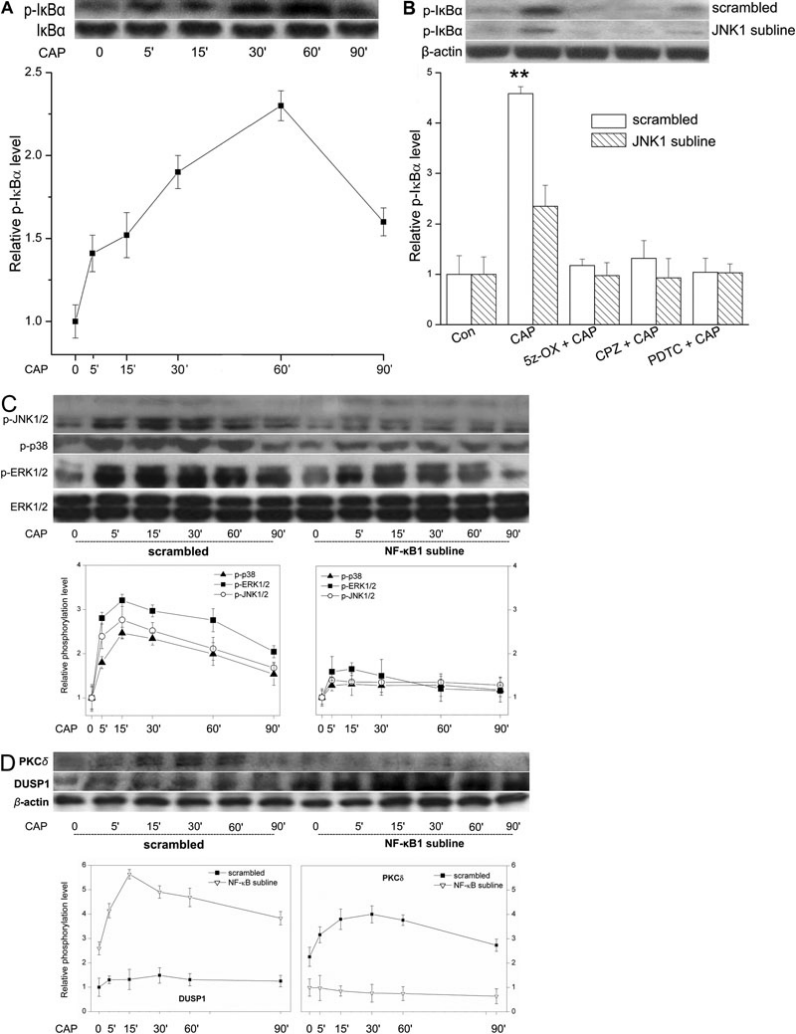
Positive feedback control of JNK1 phosphorylation by NF-κB through DUSP1. **A**: Time-dependent increases in p- IκBα. Scrambled shRNA subline was exposed to CAP (20 µM) for up to 90 min. Western blots reveal the time course of changes in p-IκBα formation, which serves as a readout of NF-κB activation. **B**: Contribution by JNK1 to IκBα phosphorylation. Western blots compare CAP (20 µM)-induced IκBα phosphorylation in scrambled shRNA and JNK1 sublines at 60 min. Preincubation with either 5z-OX (0.1 µM), CPZ (10 µM) or PDTC (50 µM) for 60 min suppressed CAP-induced IκBα phosphorylation. **C**: Positive feedback control by NF-κB of JNK1/2 activation. Loss of NF-κB activation reduces transient JNK1/2, p38, and ERK1/2 MAPK activation induced by CAP (20 µM) for up to 90 min. Summary plots contrast time-dependent patterns of MAPK activation in the scrambled shRNA subline (left) with those in NF-κB1 subline (right). **D**: Inverse relationship between changes in PKCδ and DUSP1 expression. Scrambled shRNA and NF-κB1 sublines were exposed to CAP (20 µM) as described in **B**. Summary plot (left) indicates that in the scrambled shRNA subline CAP-induced increases in PKCδ expression whereas DUSP1 remained invariant (left). Summary plot (right) reveals inverse responses by PKCδ and DUSP1 to CAP in NF-κB1 subline.

To determine whether NF-κB activation by TRPV1 is dependent on TAK1 and/or JNK1 activity, as was described for some other different receptors in other tissues [9,10], we monitored the phosphorylation status of IκBα, a protein that when unphosphorylated binds to NF-κB and stoichiometrically inhibits its activity [11]. Figure 4A shows that upon addition of CAP increases in p-IκBα formation reached after about 60 min a maximal value, which was slower than the rises in p-TAK1 expression (Figure 1A). In the JNK1 subline, relative to the scrambled subline, this CAP-induced IκBα phosphorylation was reduced only by 50%; 0.1 µM 5z-OX eliminated the remaining 50% of this response (Figure 4B) suggesting that TAK1 may also activate NF-κB through a JNK1-independent pathway.

### NF-κB activity modulates JNK1 phosphorylation status via a positive feedback loop involving DUSP1 and PKCδ

In addition to the dependence of NF-κB activation on JNK1 phosphorylation, in several other cell systems, NF-κB has been shown to increase the duration and magnitude of JNK1 phosphorylation through positive feedback control [12]. To assess the functionality of this effect in HCEC, we compared the time-dependent effects of CAP on terminal kinase phosphorylation in scrambled shRNA cells with those in the NF-κB1 subline (Figure 4C). In both cases, peak phosphorylation responses were observed at about 15 min, but the responses in this subline were diminished, suggesting that the feedback mechanism is operative. Figure 4C also indicates that similar suppression patterns were observed in p-ERK1/2 and p-p38 formation. These global declines in MAPK terminal kinase phosphorylation levels in the absence of NF-κB1 expression led us to focus attention on DUSP1, a dual specificity phosphatase, which is highly expressed in the HCEC and displays similar high affinity for all three MAPK terminal kinases [13,14]. Figure 4D shows that at 90 min post TRPV1 activation, DUSP1 expression in the NF-κB1 subline was still nearly threefold higher than that in the scrambled shRNA control cells.

Additionally, noting that in murine embryonic fibroblasts and in neurons loss of NF-κB activity has been linked to declines in PKCδ expression and rises in DUSP1 expression [15,16], we hypothesized that such a relationship also exists in HCEC. Comparative measurement of CAP on DUSP1 and PKCδ expression in the scrambled shRNA subline and NF-κB1 subline confirmed this assumption. In the control system, the results shown in Figure 4D indicate that CAP caused a nearly threefold increase in PKCδ expression whereas DUSP1 remained essentially invariant. Conversely, in the NF-κB1 subline, PKCδ remained invariant and low whereas DUSP1 expression rose rapidly by more than fourfold after 15 min.

### DUSP1 modulates TRPV1-induced IL-6/8 release through control of JNK phosphorylation

**Figure 5:**
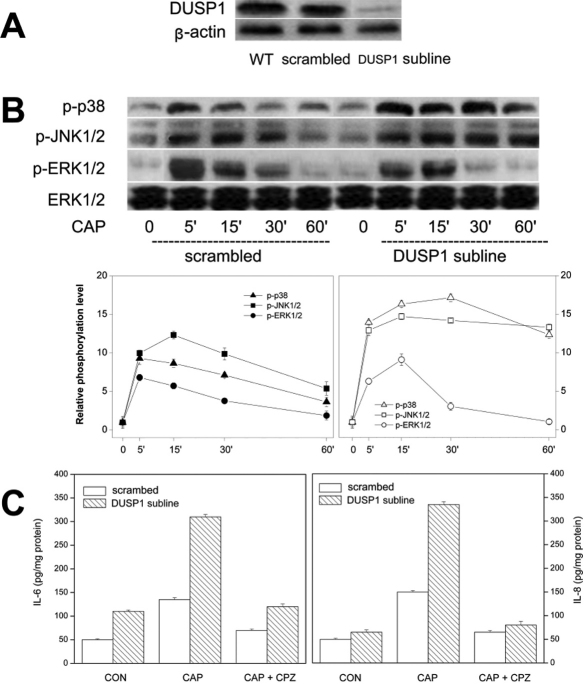
Changes in MAPK activation patterns and IL-6/8 release in DUSP1 subline. **A**: Western blot analysis of total DUSP1 protein expression in resting scrambled HCEC and DUSP1 subline. **B**: Comparison of time dependent changes in MAPK phosphorylation in scrambled shRNA and DUSP1 subline. Cells were exposed to CAP (20 µM) for indicated times. Changes are compared in p-ERK1/2, p-JNK1/2, and p38 MAPK in two different sublines. Protein loading equivalence validated based on invariant ERK1/2 expression levels. **C**: DUSP1 gene knockdown enhances CAP-induced IL-6 and IL-8 release. ELISA was performed on scrambled shRNA and DUSP1 subline after 24 h exposure to CAP (20 µM). Three independent experiments each performed in triplicate.

To assess the contribution by DUSP1 in mediating NF-κB control of JNK1 activation, CAP-induced MAPK TK activation patterns were evaluated in a DUSP1 subline displaying an 85% reduction in phosphatase expression (Figure 5A). Figure 5B shows that in the scrambled shRNA cells terminal kinase phosphorylation rapidly reached a peak within 15 min, which gradually waned during the next 60 min. On the other hand, in the DUSP1 subline, terminal kinase activation underwent larger increases at the beginning and remained invariant for a longer period particularly in the case of JNK1/2. Figure 5C shows the impact of enhanced and prolonged JNK1 phosphorylation on IL-6/8 release. After 24 h exposure to CAP, IL-6/8 release was 1.4 fold and 2.6 fold more, respectively, in the DUSP1 subline than in the scrambled cells.

## Discussion

We show here that TRPV1 mediates NF-κB activation through a JNK1-dependent and JNK1-independent pathways. Stimulation of both pathways is mediated through CAP-induced TAK1 phosphorylation. This type of control of NF-κB has been described in some other tissues [9,10,17]. JNK1 in the MAPK cascade is the only mediator of IL-6/8 release since CAP failed to induce this response in the JNK1 shRNA HCEC subline (Figure 2B). TLR2-induced IL-release is also solely dependent on JNK1 activation because in JNK1^−/−^ knockout mice and in HCEC drug blockade of JNK activation inhibited this response [7].

**Figure 6:**
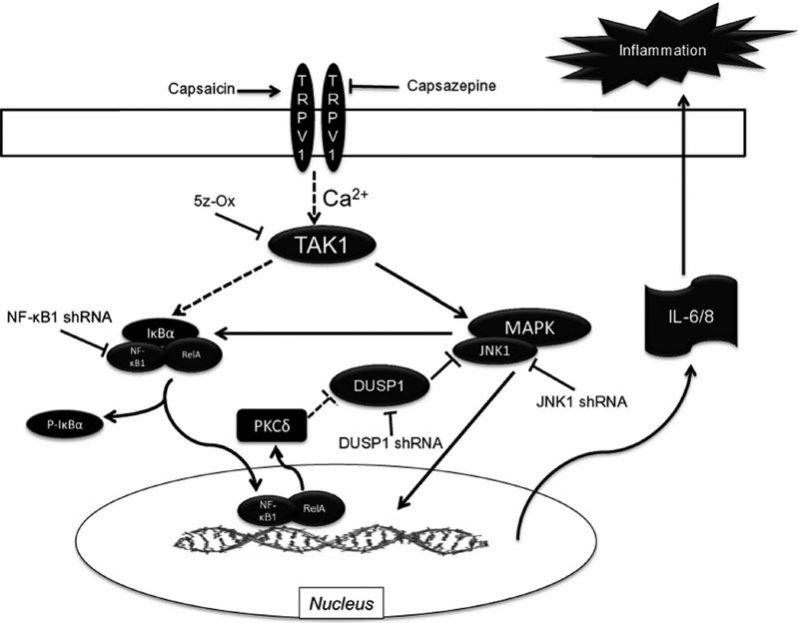
TRPV1-linked signaling pathways mediating IL-6 and IL-8 release. TRPV1 stimulation leads to TAK1-dependent JNK1 and NF-κB activation. Activated JNK1 potentiates NF-κB activation by TAK1. NF-κB provides a positive feedback control of JNK1 phosphorylation through inhibition of DUSP1 expression, which is possibly controlled by PKCδ. Activated NF-κB translocates to nucleus, along with other transcription factors activated by p-JNK1 (e.g., AP-1) to promote IL-6 and IL-8 mRNA expression. Solid line indicates established interaction. Broken line represents unproven interaction. Arrowhead means stimulation whereas hammerhead represents inhibition. Arrow pointing from TAK1 to NF-κB is broken since it is not yet known if interaction is direct or there are signaling intermediates mediating NF-κB activation by TAK1.

One of the roles of JNK1 in mediating IL-6/8 release is to activate NF-κB, a downstream essential player in this secretory process. Interleukin release is further helped or reinforced by the presence of a positive feedback loop in which NF-κB, fosters stabilization and/or prolongation of JNK1 phosphorylation since in the NF-κB1 subline CAP-induced JNK1 phosphorylation was attenuated (Figure 4C). This NF-κB to JNK1 positive feedback loop is clearly mediated by inhibition of DUSP1 activity since in the DUSP1 subline JNK1 activation was enhanced and prolonged (Figure 5B). Such suppression by NF-κB of DUSP1 expression could be dependent on NF-κB-induced increases in PKCδ protein levels. Similar NF-κB-mediated changes in DUSP1 and PKCδ expression levels have been described in embryonic fibroblasts and neurons [15,16] whereas in enterocytes, increases in NF-κB activation had a corresponding rather than an opposite effect on LPS-induced DUSP1 expression [18]. Thus, the molecular events connecting these coordinated changes and their cause and effect relationship remain to be elucidated and require independent definition in each particular system. Another remaining question is why only combined activation by CAP of the JNK1-independent and JNK1-dependent pathways is sufficient to induce IL-6/8 release. One possibility is that CAP-induced IL-6/8 release depends on c-Jun/AP-1 and NF-κB activation, which can only occur if JNK1 is sufficiently phosphorylated as a consequence of NF-κB mediated suppression of DUSP1 expression. This is tenable since AP-1 activation can occur in 15 min after exposing HCEC to a hyperosmotic stress, which is within the same time frame in which JNK1 and NF-κB activation occur (Figure 1A and Figure 4A) [19]. Our findings in the HCEC are schematically summarized in Figure 6. These findings have potential clinical relevance in identifying novel drug targets to reduce inflammation and pain resulting from exposure to environmental stresses activating TRPV1. Strategies to suppress JNK1 activation are one possibility since TRPV1-induced increases in IL-6/8 release were obviated without compromising the mitogenic response to TRPV1 activation [8]. Another alternative is to overexpress DUSP1 protein levels, which could lead to suppression of JNK1 activation and declines in IL-6/8release. This possibility is consistent with the finding that the anti-inflammatory effects of glucocorticoids are attributable to DUSP1 upregulation [20]. Direct DUSP1 drug targeting could prove to be advantageous to prolonged glucocorticoid usage which can have several ocular side effects.
